# Intima heterogeneity in stress assessment of atherosclerotic plaques

**DOI:** 10.1098/rsfs.2017.0008

**Published:** 2017-12-15

**Authors:** Ali C. Akyildiz, Lambert Speelman, Bas van Velzen, Raoul R. F. Stevens, Antonius F. W. van der Steen, Wouter Huberts, Frank J. H. Gijsen

**Affiliations:** 1 Department of Biomedical Engineering, Erasmus Medical Center, Rotterdam, The Netherlands; 2 Department of Mechanical Engineering, Delft University of Technology, Delft, The Netherlands; 3 Department of Biomedical Engineering, Maastricht University, Maastricht, The Netherlands

**Keywords:** atherosclerosis, atherosclerotic plaque stresses, finite-element analysis, heterogeneous plaque properties, global variance-based sensitivity analysis

## Abstract

Atherosclerotic plaque rupture is recognized as the primary cause of cardiac and cerebral ischaemic events. High structural plaque stresses have been shown to strongly correlate with plaque rupture. Plaque stresses can be computed with finite-element (FE) models. Current FE models employ homogeneous material properties for the heterogeneous atherosclerotic intima. This study aimed to evaluate the influence of intima heterogeneity on plaque stress computations. Two-dimensional FE models with homogeneous and heterogeneous intima were constructed from histological images of atherosclerotic human coronaries (*n* = 12). For homogeneous models, a single stiffness value was employed for the entire intima. For heterogeneous models, the intima was subdivided into four clusters based on the histological information and different stiffness values were assigned to the clusters. To cover the reported local intima stiffness range, 100 cluster stiffness combinations were simulated. Peak cap stresses (PCSs) from the homogeneous and heterogeneous models were analysed and compared. By using a global variance-based sensitivity analysis, the influence of the cluster stiffnesses on the PCS variation in the heterogeneous intima models was determined. Per plaque, the median PCS values of the heterogeneous models ranged from 27 to 160 kPa, and the PCS range varied between 43 and 218 kPa. On average, the homogeneous model PCS values differed from the median PCS values of heterogeneous models by 14%. A positive correlation (*R*^2^ = 0.72) was found between the homogeneous model PCS and the PCS range of the heterogeneous models. Sensitivity analysis showed that the highest main sensitivity index per plaque ranged from 0.26 to 0.83, and the average was 0.47. Intima heterogeneity resulted in substantial changes in PCS, warranting stress analyses with heterogeneous intima properties for plaque-specific, high accuracy stress assessment. Yet, computations with homogeneous intima assumption are still valuable to perform sensitivity analyses or parametric studies for testing the effect of plaque geometry on PCS. Moreover, homogeneous intima models can help identify low PCS, stable type plaques with thick caps. Yet, for thin cap plaques, accurate stiffness measurements of the clusters in the cap and stress analysis with heterogeneous cap properties are required to characterize the plaque stability.

## 1. Introduction

Atherosclerotic plaque rupture in coronary and carotid arteries is recognized as the primary cause of cardiac and cerebral ischaemic events [1–4]. The rupture of the plaque cap, which separates the lipid-rich necrotic core from the blood, triggers thrombotic processes and subsequently leads to on-site restriction of the blood flow or distal embolization [5]. For effective surgical treatment planning, predicting the rupture risk of an atherosclerotic plaque is of great importance; however, currently, there are no reliable methods for rupture risk assessment.

From a biomechanical perspective, cap rupture is the failure of the plaque material. The plaque loses its structural integrity when it cannot withstand the mechanical loading applied on it. The mechanically most prominent loading in the vascular system is the blood pressure, resulting in structural stresses in plaques. High plaque stresses were shown to strongly correlate with the location of plaque rupture [6–8].

Plaque stresses can be computed with finite-element (FE) techniques [9,10] and as such, FE plaque modelling holds great potential for plaque rupture risk assessment. The predictive accuracy of FE models depends strongly on the accurate representation of mechanical behaviour of plaque components [11,12]. The mechanically relevant plaque components are the arterial wall layers (adventitia and media), lipid-rich necrotic core, calcifications and intima [13]. The intima particularly plays an important role in biomechanical plaque modelling, because the cap is part of the intima and intima material properties significantly affect the computed plaque stresses [9,11,12].

In FE modelling of atherosclerotic plaques, the mechanical properties of the intima have been traditionally assumed to be homogeneous [14]. However, histological analyses of atherosclerotic plaques have demonstrated their heterogeneous structural composition, primarily consisting of collagen fibres, smooth muscle cells, inflammatory cells, fatty material and extracellular matrix [13,15,16]. As the mechanical properties of a biological tissue are determined by its structural micro-constituents, the heterogeneous composition of the intima is highly likely to result in a strong heterogeneity in its mechanical properties. A recent experimental study provided clear evidence for this by demonstrating a wide range of local stiffness values from atherosclerotic intima samples *ex vivo* [17]. Hence, the validity of the homogeneity assumption for the mechanical properties of the intima in plaque FE models and the effect of heterogeneous material behaviour of intima on plaque stresses require further investigation.

The current study aimed to evaluate the influence of atherosclerotic intima heterogeneity on plaque stresses. FE models were generated using realistic plaque geometries from histology images of human coronary arteries with advanced stage atherosclerotic plaques. Heterogeneous intima was constructed by subdividing it in an automated, objective manner into subregions, which are anticipated to show distinct mechanical behaviour. By incorporating intima material heterogeneity in the FE computations and performing a parametric study on local material properties with approximately 1200 simulations, the stress results from plaque models with heterogeneous intima were compared to the ones with traditional homogeneous intima assumption. Moreover, through a global variance-based sensitivity analysis, applied for atherosclerotic plaque stress analysis for the first time, the contribution of the intima heterogeneity to the stress variations was assessed.

## 2. Methods

### 2.1. Plaque geometry from histology

**Figure 1.**
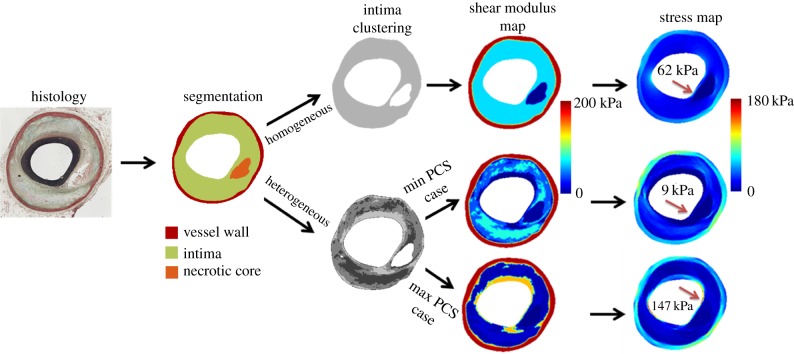
Illustration of how the FE models with homogeneous and heterogeneous intima were generated. First, the vessel wall, intima and lipid-rich necrotic core were segmented on the histology image. For the homogeneous model, the intima contained a single cluster. For heterogeneous intima models, the intima was further subdivided into four clusters based on the greyscale histology image pixel intensities, by using *k*-means clustering algorithm. Different shear modulus values were assigned to the intima clusters to generate mechanical heterogeneity in the intima. The shear modulus maps and simulated stress maps are shown for the homogeneous intima case and the heterogeneous cases with minimum and maximum PCS values.

Plaque geometries were obtained from the histological cross sections (*n* = 12) of six atherosclerotic human coronary segments from four patients who died of cardiovascular disease. For histology, the segments were fixed at an intraluminal pressure of 100 mmHg to prevent collapse of the lumen. The vessel wall (adventitia and media), lipid-rich necrotic core and intima regions were segmented manually on Movat's pentachrome-stained histological images, obtained with a magnification of 20× (figure 1). This segmentation provided the plaque geometries for the two-dimensional FE models with homogeneous intima.

### 2.2. Heterogeneous intima geometry

Histology provides subcellular level resolution on the tissue structure, whereas FE modelling requires geometric information on the macroscopic, continuum level. To obtain plaque geometries for FE simulations at macroscopic level with a representative heterogeneity, the intima region was further subdivided into four clusters based on the histological information (figure 1). The number of the clusters was chosen to be four, so that the material heterogeneity of plaque intima would be represented taking into account the presence (or absence) of the mechanically relevant microstructural constituents of plaque intima, such as collagen fibres and smooth muscle cells.

First, the histology images were converted into greyscale images and all plaque components other than the intima were masked. Then, four clusters within the intima region were generated based on the greyscale intensities of the pixels by using *k*-means clustering algorithm [18]. The working principle of this iterative clustering algorithm was as follows: first, four random values within the greyscale intensity range of the intima region were defined as the mean intensity values for the four clusters and each pixel within the intima was assigned to the cluster with the mean value closest to the pixel intensity. Then, new cluster means were calculated from the intensity values of the pixels in the clusters and the pixels were redistributed to the clusters with the new means. This process was repeated iteratively until there was no change in the cluster means. After generating the four intima clusters, erosion and merging steps were performed to remove isolated cluster islands of pixels and to obtain representative intima heterogeneity for the FE simulations.

### 2.3. Material properties of homogeneous and heterogeneous intima

The material properties of the homogeneous intima and the four clusters in the heterogeneous intima models were based on a recent experimental study [17], which investigated the local mechanical properties of atherosclerotic plaques. In that study, the local plaque properties were described with the incompressible neo-Hookean material formulation, for which the strain energy density potential, *W*, is defined as *W* *=* *C*(*I*_1_–3). Here, *C* is the material constant, namely the shear modulus, and *I*_1_ is the first invariant of the left Cauchy–Green deformation tensor. The values of the shear modulus reported in the study ranged from 1 to 149 kPa [17].

Similarly, neo-Hookean material models were used in the current study for the intima properties. For homogeneous intima, the mean of the reported shear modulus values (=75 kPa) was employed. For the heterogeneous intima models, a shear modulus value from the reported range was assigned to each cluster. As the exact mechanical properties of the intima clusters were unknown, 100 different shear modulus combinations for the four clusters were generated to cover the reported range. The combinations were generated by using Latin hypercube sampling method [19], which enabled a uniform sampling in the input space. Subsequently, Gaussian smoothing was applied to the shear modulus maps to avoid sharp material transitions in the FE models (figure 1).

### 2.4. Finite-element plaque models

The two-dimensional FE models of the 12 plaque cross sections with homogeneous and heterogeneous intima were created from the histology segmentation and intima clustering as described above. The FE analyses were performed with ABAQUS (Version 6.14, Dassault Systemes Simulia Corp., Providence, RI, USA). Large deformation formulation and plane strain assumption were used in the simulations. Three-node and four-node, linear, plane strain, hybrid elements with constant pressure (CPE3H and CPE4H) were used. After conducting mesh sensitivity analyses, the FE models had approximately 20 k elements. Similar to the intima, neo-Hookean material models were employed for the vessel wall and lipid pool components, with shear moduli of 200 and 0.5 kPa, respectively [10]. The rigid body motion in the models was constrained by adding a gel-like, highly compressible, compliant (shear modulus = 0.001 kPa) section surrounding the cross sections and encastering the circular outer edge of this section. As the histological images were acquired from the plaque pressure fixed at 100 mmHg, the initial stresses due to this intraluminal pressure at the initial geometry were computed using a previously developed technique first [10]. Then, an additional intraluminal pressure of 20 mmHg was imposed as the final loading condition to reach a systolic pressure of 120 mmHg and maximum principal stress values were extracted. The cap region of the plaques was identified in the models, and the peak cap stress (PCS) in the homogeneous (*n* = 1 per plaque) and heterogeneous (*n* = 100 per plaque) FE models was acquired. Heterogeneous intima model PCS results were compared to the homogeneous intima results and the variations in the heterogeneous model PCS results were analysed.

### 2.5. Global variance-based sensitivity analysis

A global variance-based sensitivity analysis was used to assess the influence of the shear modulus of the intima clusters (input parameters) on the PCS results (output parameter) of the heterogeneous intima FE simulations. A global method was chosen as it required no assumptions regarding the model's linearity, monotonicity or additivity [20]. In the sensitivity analysis, by using Sobol decomposition, the PCS variation was apportioned to the individual input parameters and the interactions between the parameters. The individual input parameter contributions and interaction contributions were estimated using a metamodelling approach based on the generalized polynomial chaos expansion (gPCE) [21–23]. The gPCE method approximated the original model output by means of a finite sum of orthogonal multi-variate polynomial basis functions that were functions of the model input parameters [24]. Legendre polynomials were used as the basis functions because of the uniform distribution of the input parameters [25]. An adaptive scheme was used to construct the metamodel, where a new basis function was added in case this improved metamodel's descriptive capability. The procedure was terminated when the predictive error, computed based on a leave-one-out cross-validation, reached 0.001. In the end, the so-called main sensitivity indices were obtained from the sensitivity analysis, which quantified the individual input parameter contributions. It should be noted that the sum of the main indices and the interaction terms equals 1. For more information about the sensitivity analysis methodology, the reader is referred to Quicken *et al.* [23].

## 3. Results

Incorporating intima heterogeneity in the FE analysis induced variation in the computed plaque stresses. The right-hand side of figure 1 exemplifies this finding by demonstrating the shear modulus maps and plaque stresses of a plaque for the homogeneous intima model (top row) and heterogeneous models with the lowest PCS (middle row) and highest PCS (bottom row). This plaque had a PCS of 62 kPa for the homogeneous model, whereas for the heterogeneous models, the PCS ranged from 9 to 147 kPa. Besides the approximately 16-fold difference between the PCS values of the two heterogeneous models, also the PCS location (arrows in figure 1) differed between the models and shifted from one shoulder of the cap to the other one.

**Figure 2.**
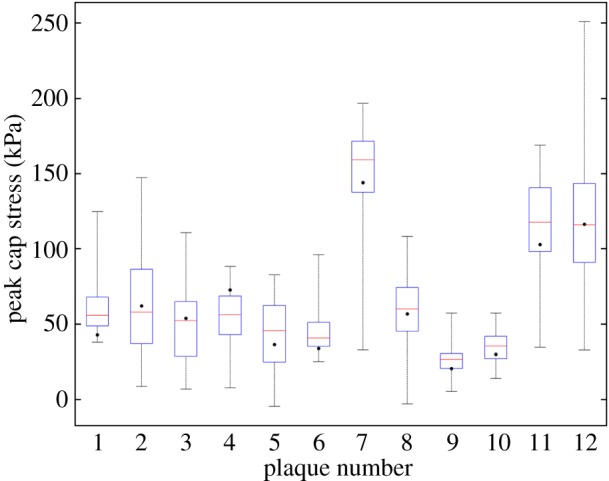
PCS results (kPa) of the heterogeneous intima models and the homogeneous intima models (black dots) for all 12 plaques. The red lines depict the median values, the blue boxes the interquartile range and the vertical lines the entire ranges of the PCS values from the heterogeneous intima models per plaque.

**Figure 3.**
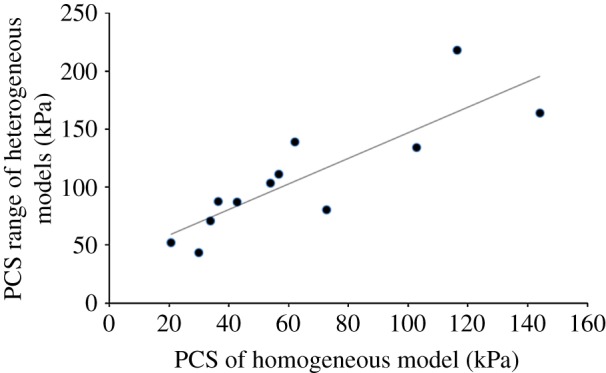
Correlation between the PCS range of the heterogeneous intima models and the PCS of the homogeneous intima model.

In total, 1212 FE simulations (one for homogeneous and 100 for heterogeneous intima per plaque) were performed. The PCS results for all 12 plaques are summarized in figure 2. The median PCS values of the heterogeneous models (red lines in figure 2) varied between 27 and 160 kPa. The PCS range per plaque (vertical lines in figure 2) had a minimum value of 43 kPa (plaque no. 10) and a maximum value of 218 kPa (plaque no. 12). For the majority of the plaques (9 of 12), the homogeneous model PCS (black dots in figure 2) was within the interquartile range (blue boxes) of the heterogeneous model results. On average, the absolute difference between the homogeneous model PCS values and the median PCS values of heterogeneous models was 14%. A strong correlation (*R*^2^ = 0.72, figure 3) was found between the PCS of the homogeneous models and the PCS range of the heterogeneous models: the higher the PCS of the homogeneous model was, the larger the PCS variation of the heterogeneous models was.

**Figure 4.**
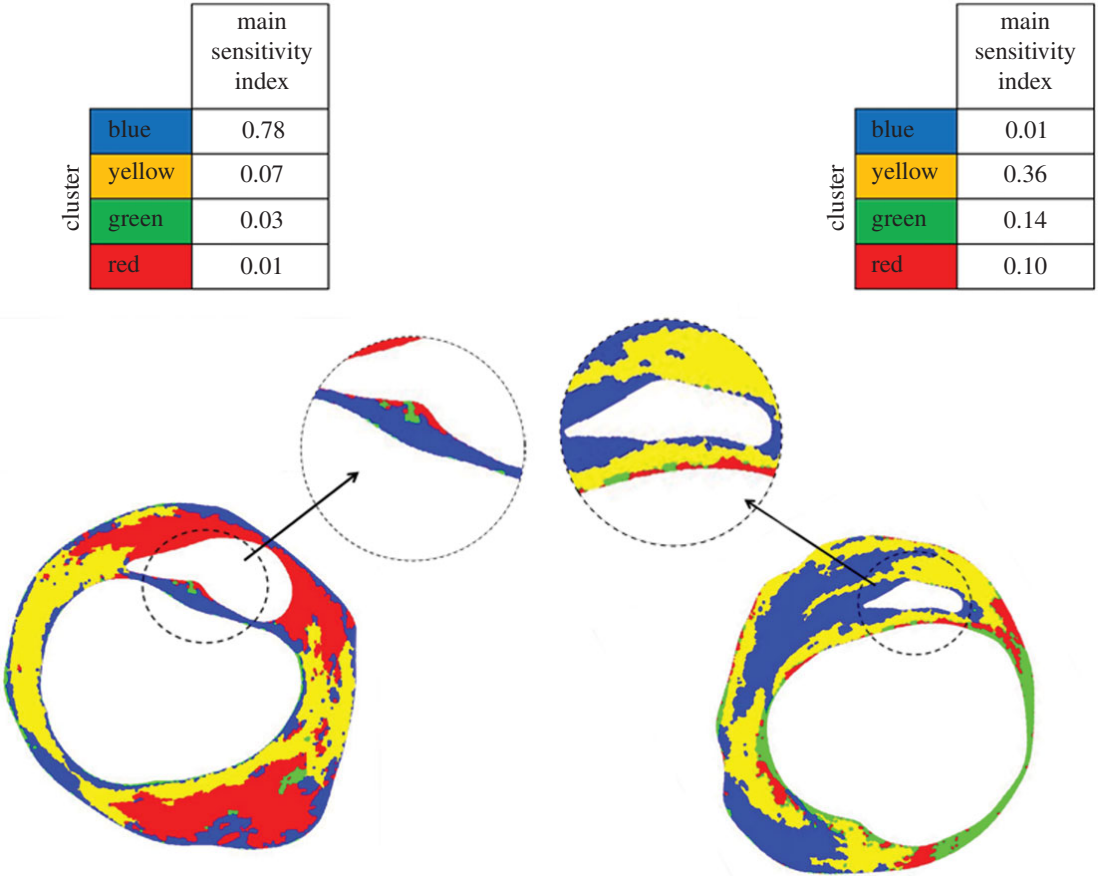
A case with a high maximum main index (left, plaque no. 5) and a case with a low maximum main index (right, plaque no. 6) from the global variance-based sensitivity analysis.

The variance-based sensitivity analyses revealed how the PCS variation in a plaque was affected by the variation of the stiffness of the intima clusters. The case of plaque no. 5 is given as an example in the left panel of figure 4. For this plaque, the blue cluster had the highest main sensitivity index with a value of 0.78, indicating that using the exact material properties of this cluster in the stress computations would reduce the variation in PCS results by 78%. The yellow cluster followed the blue cluster, with a main sensitivity index of 0.07 and the indices of the green and red clusters were lower than 0.05. Some plaques showed lower highest main sensitivity index, such as plaque no. 6 (figure 4, right panel). For this plaque, the highest main index was 0.36 (yellow cluster), and the second and third highest main indices were 0.14 and 0.10. The lowest main index was 0.01. It is also to be noted that the sum of the main sensitivity indices for this plaque was much lower than 1, indicating strong interaction terms in the metamodel. Overall, the highest main sensitivity index per plaque varied from 0.26 to 0.83, and with an average of 0.47. For seven out of the 12 plaques, the cluster with the highest main sensitivity index was the largest cluster in the thinnest region of the cap.

## 4. Discussion

For planning preventive treatment strategies, it is of great importance to accurately perform rupture risk stratification of atherosclerotic plaques and to correctly identify if a plaque is a low risk, stable type or a high risk, vulnerable type. Previously, high cap stresses were shown to strongly correlate with the plaque rupture location [6–8], hence high PCS is recognized as an indicator of a vulnerable plaque and low PCS as the indicator of a stable plaque. Stress computations in atherosclerotic plaques have been traditionally carried out with the assumption of homogeneous material behaviour of the intima. However, a recent experimental study provided evidence for the mechanical heterogeneity of the atherosclerotic intima [17]. In this study, the influence of heterogeneous intima properties on PCS was assessed by incorporating the full range of intima material heterogeneity in plaque stress computations for the first time.

The changes in PCS due to intima heterogeneity were substantial (PCS range/median PCS = 167% on average), implying that heterogeneous intima material behaviour is required in the FE simulations for plaque-specific, precise stress assessment. Yet, the interquartile PCS ranges of the heterogeneous models were relatively moderate (PCS range/median PCS = 49% on average). Moreover, in most plaques, the homogeneity assumption for the intima material behaviour resulted in a PCS value within the interquartile PCS range, which slightly differed (14% on average) from the median PCS of the heterogeneous models. These findings suggest that despite the heterogeneous nature of atherosclerotic intima, homogeneous intima models are still valuable to assess the influence of plaque morphology on plaque stresses and to perform sensitivity analyses or parametric studies for testing the effect of plaque geometry on PCS and identifying geometric fingerprints of stable and vulnerable plaque types [9,12,26,27].

The strong positive correlation observed for this study sample between the homogeneous model PCS and the heterogeneous model PCS range suggests the predictive potential of the homogeneous models for the heterogeneity-driven PCS variability. In the case of a low PCS from homogeneous intima FE analysis, the PCS variability in the heterogeneous intima models was low. This implies that it is unlikely for low PCS plaques to have a high PCS due to heterogeneous intima properties. Low PCS is usually associated with a thick cap [9,12]. It was also demonstrated that precise cap thickness measurement in plaques with a thick cap is not crucial to identify them as low PCS, stable plaques; hence low resolution, non-invasive techniques such as magnetic resonance imaging are sufficient [28]. So, both from the imaging and modelling viewpoints, identification of thick cap, stable plaques is feasible.

High PCS is majorly associated with a thin cap [9,12]. To precisely determine if a thin cap plaque will have a high PCS, cap thickness has to be accurately measured. In this respect, optical coherence tomography (OCT) is an attractive candidate as it enables high-resolution (approx. 10 µm) plaque and cap imaging. A plaque cap might be structurally fairly homogeneous, such as the fibrous caps, which are mainly composed of collagen fibres [13]. Based on the homogeneous cap stiffness assumption, we previously demonstrated that not only the cap thickness but also the cap stiffness has a significant influence on the PCS results [9,12]. In the case of a homogeneous cap, optical coherence elastography (OCE), which uses the superior spatial resolution of OCT, might offer a possibility to measure the stiffness of the thin homogeneous caps. However, the cap region might be structurally heterogeneous as well as the rest of the atherosclerotic intima. This was also the case for all 12 plaque morphologies investigated in the current study, including the ones with a cap thickness of as low as 35 µm. The current study demonstrated that the intima heterogeneity might amplify the PCS in these high-risk plaques. Although the sensitivity analysis showed main indices as high as 0.83, the average value of the highest main index was 0.47. This implies that it is not possible to sufficiently reduce the uncertainty in PCS results for risk stratification by measuring the stiffness of only one cluster, but it is required to determine the heterogeneous properties of the cap and plaque. While OCE is an attractive option for measuring stiffness of homogeneous thin cap structures, its spatial resolution might be insufficient to assess heterogeneous cap properties. Hence, accurate PCS assessment of such high-risk plaques with thin heterogeneous caps requires further development in imaging and tissue characterization techniques. Another limitation of OCT, when used for PCS assessment, is the limited penetration depth, preventing accurate delineation of the backside of larger necrotic cores [29]. To overcome this limitation, one can make use of geometrical models that allow the estimation of the necrotic core thickness [30], or combine OCT with other imaging techniques like intravascular ultrasound.

A few limitations of this study have to be noted. First, atherosclerotic intima was assumed to be composed of four subregions/clusters that show distinctly different mechanical behaviour. The rationale for this was that the macroscopic regional material behaviour in atherosclerotic intima depends on the presence (or absence) of the mechanically relevant microstructural constituents, namely collagen fibres and smooth muscle cells. Hence, the number of four clusters was considered to be reasonable. Moreover, intima subregion segmentation was based on pixel intensity of greyscale histology images; hence, the clusters did not directly reflect the constituents of the intima. Despite these limitations, the employed approach resulted in a representative segmentation of the heterogeneous intima for the study goal of investigating the effect of intima heterogeneity of plaque stresses. Secondly, as the exact material behaviour of the clusters in the heterogeneous intima models and the exact relationship of image intensity to material properties were unknown, experiment-based but arbitrary material properties were assigned to the clusters, preventing us from computing the exact PCS of the plaques investigated. Yet, by testing a substantial number of material behaviour combinations (*n* = 100), the entire range of local material properties for atherosclerotic intima, reported by a recent experimental study, was covered, including the ‘worst-case scenarios’ for the variation in PCS. Heterogeneous intima material properties used in the FE simulations were from a previous experimental study [17], which, to the best knowledge of the authors, is the only one capturing and reporting local atherosclerotic intima properties. In this experimental study, indentation loading was performed in the longitudinal direction of the artery. Although this does not mimic the physiological loading condition in the cardiovascular system perfectly, indentation loading results in transverse deformation in the tissue, in this case, in the circumferential direction of the artery, which is the dominant physiological loading direction. Moreover, indentation testing enables studying local properties, whereas the standard testing techniques only provide average/gross tissue properties. The possible anisotropic material behaviour of the plaque components was not simulated as detailed experimental data are still not available. Previously, we have shown that taking initial stresses into account in FE plaque simulations might have a substantial effect on PCS results, ranging from −40% to +28% [10]. Therefore, initial stresses were incorporated in the computations. However, residual stresses [31] were not, because there is currently no reliable means of estimating them. A previous study of our group demonstrated −33% to +48% difference in PCSs between full three-dimensional and two-dimensional simulations [32]. FE simulations in the current study are two-dimensional as the histology images, which provide high detail, high-resolution plaque information, cannot provide three-dimensional plaque geometry. Yet, the range of the computed PCSs in this study due to intima heterogeneity is much larger than the ones when initial stresses are included and three-dimensional simulations are performed. This implies that the potential impact of local intima heterogeneity cannot be ignored for absolute PCS computations. The effect of residual stresses, anisotropic material behaviour, three-dimensional FE modelling combined with intima heterogeneity warrants future research; however, the authors do not expect any changes in the main conclusions of the current study.

To the best knowledge of the authors, this is the first study that included intima heterogeneity in plaque stress analyses. The generated dataset provides valuable insight in the variation of the computed cap stresses due to the intima heterogeneity. By performing a substantial number of computations, clear numerical evidence has been provided for the significant influence of heterogeneous intima properties on PCSs.

## Data accessibility

This article has no additional data.
